# Supracardiac Total Anomalous Pulmonary Venous Connection in Adolescence

**DOI:** 10.7759/cureus.47392

**Published:** 2023-10-20

**Authors:** Firdoos Ahmad Mir, Satyapriya Mohanty, Arvind Pandey, Asiya Naqashbandi, Debasish Das

**Affiliations:** 1 Cardiothoracic Surgery, Sher-i-Kashmir Institute of Medical Sciences, Srinagar, IND; 2 Cardiothoracic and Vascular Surgery, All India Institute of Medical Sciences, Bhubaneswar, Bhubaneswar, IND; 3 Cardiothoracic Surgery, Banaras Hindu University Institute of Medical Sciences, Varanasi, IND; 4 Pediatrics, District Hospital Kulgam, Kulgam, IND; 5 Cardiology, All India Institute of Medical Sciences, Bhubaneswar, Bhubaneswar, IND

**Keywords:** adolescence, tapvc, supracardiac, repair, surgical

## Abstract

Background

Supracardiac total anomalous pulmonary communication (TAPVC) constitutes a rare congenital cardiac anomaly. Most babies with supracardiac TAPVC are diagnosed in infancy and undergo complete surgical repair during infancy. Delayed presentation of supracardiac TAPVC is rare, and the surgical outcomes are not well known. This retrospective study was conducted to determine the presentation and surgical outcome of supracardiac TAPVC among adolescents, which constitutes an extremely rare subgroup of TAPVC.

Methodology

This retrospective analysis was conducted among 15 adolescent patients with supracardiac TAPVC who underwent surgical repair in the cardiothoracic surgery department of a tertiary care center in India. This study aimed to assess the intraoperative, postoperative, immediate, early, and late outcomes of adolescent patients with supracardiac TAPVC who had undergone surgical repair between 2010 and 2014 in a tertiary care center in India.

Results

The study included 15 patients with a diagnosis of isolated supracardiac TAPVC. A mild degree of cyanosis was present in eight patients, recurrent episodes of lower respiratory tract infections were present in five patients, and dyspnea (New York Heart Association I/II) was noted in 12 patients. Mean oxygen saturation was 92% (range = 85-93%), and mean pulmonary artery pressure was 24 mmHg (range = 15-50 mmHg). After median stenotomy, a wide anastomosis was made between the common pulmonary venous chamber and the posterior wall of the left atrium. A fenestration was made in the Dacron patch in three patients who had raised pulmonary vascular resistance (PVR) preoperatively. Twelve patients were weaned off cardiopulmonary bypass (CPB) with minimal inotropic support. Three patients who had high preoperative PVR had difficulty in weaning from CPB. The mean CPB and cross-clamp time was 75 ± 12 minutes and 58 ± 9 minutes, respectively. Atrial fibrillation was noted in five (33.3%) patients in the early postoperative period, and three (20%) patients had pulmonary artery hypertensive crises postoperatively. There was no superficial or deep sternal wound infection in the postoperative period. Mild and moderate right ventricular dysfunction was present in four (26.67%) and two (13.3%) patients, respectively, in the postoperative period. On two-dimensional echocardiography during follow-up at the end of one year, there was no gradient across the anastomosis, and pulmonary artery pressure was normal in all patients.

Conclusions

Surgical repair of supracardiac TAPVC in adolescence has an excellent outcome. Survival of patients with supracardiac TAPVC until adolescence depends on the presence of a dilated vertical vein and a large atrial septal defect facilitating unobstructed pulmonary venous flow. The aim of the surgical repair should be to create a wide anastomosis between the left atrium and the pulmonary venous chamber which should be bigger than the size of the mitral valve orifice indexed to the body surface area as it would amount to no or negligible anastomotic gradient postoperatively.

## Introduction

The total anomalous pulmonary venous connection (TAPVC), with an incidence of 0.058 to 0.083 in 1,000 live births, accounts for 1% to 3% of congenital heart diseases. The majority of these patients present in the neonatal period with decompensated heart failure due to pulmonary venous return obstruction [1]. The natural history of the TAPVC reveals that only 20% of these patients survive beyond infancy without treatment [2]. Survival of patients with supracardiac TAPVC beyond infancy depends on the presence of large-sized vertical veins, large-sized atrial septal defect (ASD), and low pulmonary vascular resistance (PVR) [3]. These patients present with mild cyanosis and mild exercise intolerance as they grow. In the second decade of life, the development of pulmonary vascular disease leads to an increase in cyanosis secondary to the development of Eisenmenger syndrome [4,5]. These findings suggest the feasibility of successful repair of supracardiac TAPVC in adolescent patients. To date, there is limited experience of successful surgical repair of supracardiac TAPVC in adolescents. In this study, we retrospectively analyzed the outcomes of surgical management of supracardiac TAPVC presenting in the adolescent age group and assessed the factors affecting the surgical outcomes.

## Materials and methods

This retrospective study was conducted at the Department of Cardiothoracic and Vascular Surgery of Vardhman Mahavir Medical College and Safdarjung Hospital, India from August 2010 to March 2014. This study aimed to assess the intraoperative, postoperative, immediate, early, and late outcomes of adolescent patients with supracardiac TAPVC who had undergone surgical repair between 2010 and 2014. Data from all patients including clinical case sheets and operative notes were obtained after obtaining approval from the Institutional Ethics Committee of Vardhman Mahavir Medical College and Safdarjung Hospital (approval number: IEC/vmmc-sjh/PG thesis/11) and informed patient consent.

The study included 15 adolescent patients (10-14 years) with supracardiac TAPVC who had undergone surgical repair during the study period. Patients who required surgical repair of associated lesions besides supracardiac TAPVC were excluded from the study.

Baseline demographic data, detailed history with special emphasis on recurrent respiratory tract infection in early childhood, clinical profile including baseline oxygen saturation (SpO_2_) and presence of cyanosis, and preoperative pulmonary venous resistance (PVR) in cardiac catheterization, which is the most important parameter before surgery, were obtained for each individual. Each individual patient’s operative note was analyzed with special emphasis on the technical aspect of vertical vein ligation and the need for fenestrated ASD patch closure in the presence of high PVR. Intraoperative parameters included mean cardiopulmonary bypass (CPB) and cross-clamp time. Postoperative parameters included the need for high ionotropic support, difficult weaning, mean ionotropic support time, mean ventilatory support time, postoperative atrial fibrillation, development of the pulmonary hypertensive crisis, renal failure, acidosis, the need for re-exploration, total hospital stay, and postoperative mortality. Discharge echocardiography parameters with an emphasis on the presence of right ventricular systolic dysfunction and gradient across the pulmonary vein were analyzed in each patient. Follow-up records of each patient at one year which included oxygen saturation, New York Heart Association (NYHA) functional class, and echocardiographic gradient across the pulmonary vein were also analyzed to assess the late outcomes of surgical repair of supracardiac TAPVC in adolescents.

The continuous variables were expressed as mean ± SD, and the categorical variables were expressed as median and interquartile range. Simple descriptive statistics were used to analyze the data via SPSS software version 2021 (IBM Corp., Armonk, NY, USA).

## Results

**Figure 1:**
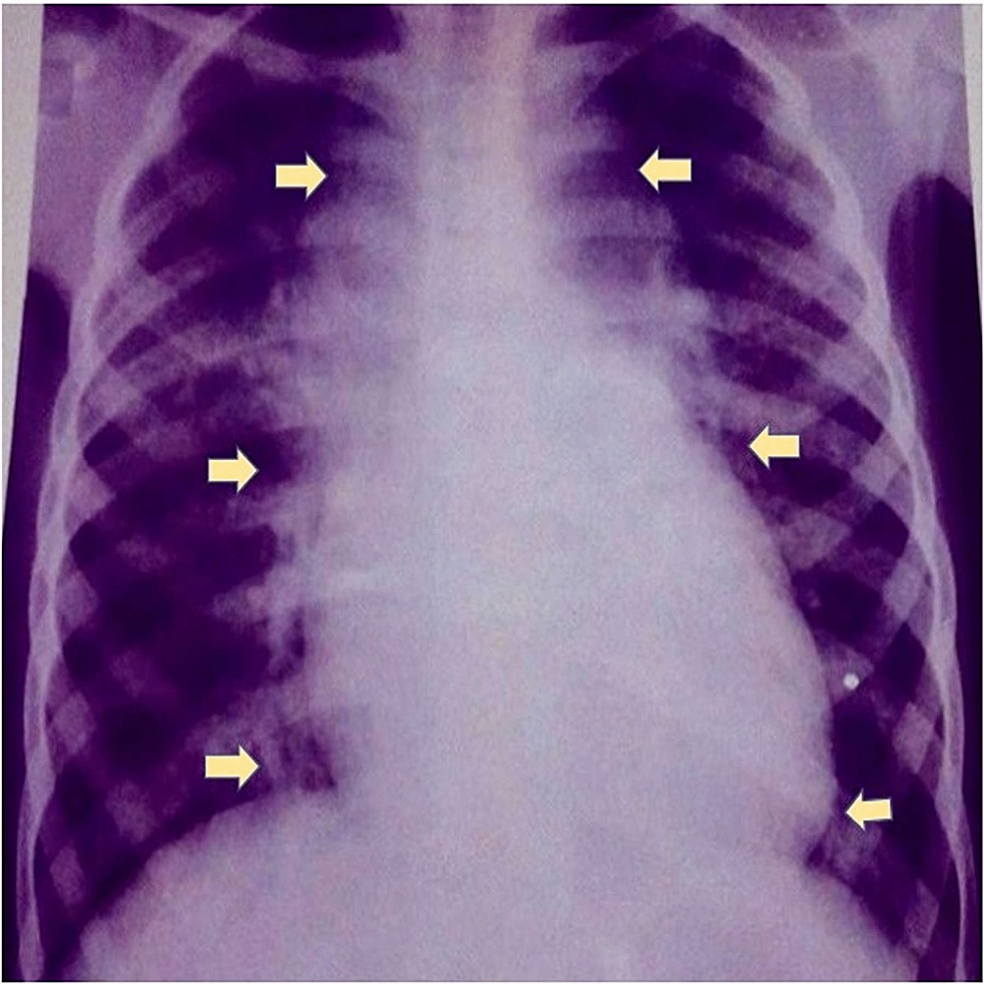
Appearance of cardiac shadow on chest X-ray posteroanterior view in total anomalous pulmonary communication.

**Figure 2:**
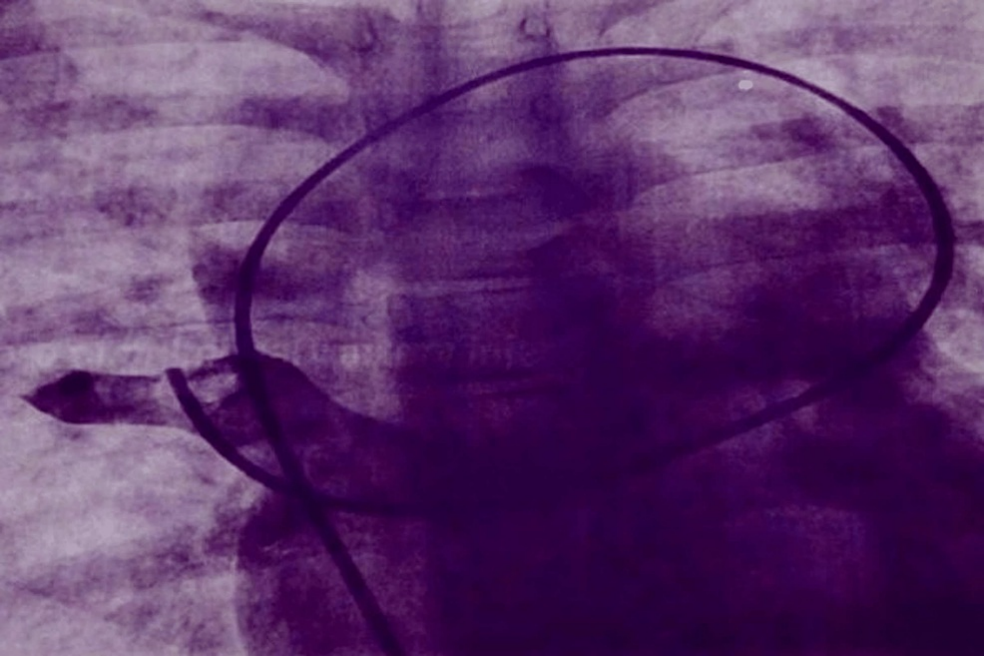
Catheter course of supracardiac total anomalous pulmonary communication.

This study included 15 patients (10 males and five females) with a mean age of 10.5 ± 0.5 years and an average weight of 25 ± 1 kg with a diagnosis of isolated supracardiac TAPVC. A mild degree of cyanosis was present in eight patients, recurrent episodes of lower respiratory tract infections were present in five patients, and dyspnea (NYHA I/II) was noted in 12 patients (Table 1). The chest X-rays revealed cardiomegaly with the classical snowman appearance (Figure 1). The echocardiographic examination revealed a large and dilated venous confluence (pulmonary venous chamber) with a dilated vertical vein, a large ASD, and biventricular hypertrophy with right ventricular volume overload. Cardiac catheterization was performed for all patients. Mean oxygen saturation was 92% (range = 85-93%), mean pulmonary artery pressure was 24 mmHg (range = 15-50 mmHg), and mean systemic pressure (systolic) was 108 mmHg (range = 92-138 mmHg) (Table 2). Levophase of pulmonary artery injection showed pulmonary venous drainage into the dilated vertical vein and the catheter course of supracardiac TAPVC (Figure 2). Pulmonary vascular resistance was 7-9 Wood units in three (20%) patients, which had a demonstrable fall to <7 Wood units on administering 100% oxygen.

**Table 1:** Baseline variables of study subjects. PVR: pulmonary vascular resistance

Baseline variables	Results (n = 15)
Males, N (%)	10 (66.66)
Females, N (%)	5 (33.33)
Mean age, years	10.5 ± 0.5
Patients with mild cyanosis, N (%)	8 (53.33)
Patients with a history of recurrent lower respiratory tract infection, N (%)	5 (33.33)
Mean oxygen saturation, %	92 (85–93)
Mean pulmonary artery pressure, mmHg	24 (15–50)
Mean systolic pressure, mmHg	108 (92–138)
Patients with high PVR, N (%) (>6 wood units)	3 (20%)

**Table 2:** Intraoperative, postoperative, immediate, early, and late variables of 15 adolescent TAPVC patients who had undergone surgical repair. TAPVC: total anomalous pulmonary communication; CPB: cardiopulmonary bypass; ASD: atrial septal defect; PVR: pulmonary vascular resistance; ICU: intensive care unit; NYHA: New York Heart Association

Intraoperative, postoperative, immediate, early, and late variables	Number of patients (%)
Mean CPB time (minutes)	75 ± 12
Mean cross-clamp time (minutes)	58 ± 9
Vertical vein ligation	14 (93.33)
Fenestrated ASD closure due to high PVR	3 (20%)
Removal of fenestrated ASD closure	1 (6.66%)
Difficult weaning due to high PVR	3 (20)
Need for high ionotropic support due to preoperative high PVR	2 (13.33)
Reexplorations	0 (0)
Atrial fibrillation	5 (33.33)
Pulmonary hypertensive crisis	2 (13.3)
Low cardiac output syndrome	1 (6.66)
Metabolic acidosis	1 (6.66)
Mean ventilatory support time (hours)	32 ± 5.3
Mean ionotropic support time (days)	2.1 ± 1.2
Mean ICU stay	2.5 ± 0.8
Mean hospital stay	8 ± 1.2
Postoperative pulmonary valve gradient	0 (0)
Postoperative mild right ventricular systolic dysfunction	4 (26.67)
Postoperative moderate right ventricular systolic dysfunction	2 (13.3)
NYHA functional class I and II at 1 year	12 (0.8)

Table 2 presents the intraoperative, postoperative, immediate, early, and late outcome parameters of 15 adolescent TAPVC patients who had undergone surgical repair in the cardiac surgery department of the tertiary care hospital. The median sternotomy approach was used in all patients. CPB was instituted using the ascending aorta and direct bicaval cannulation after full heparinization. The vertical vein was identified, looped, and snugged. The aorta was cross-clamped, and root cardioplegia was injected with moderate systemic hypothermia. The right pleural cavity was opened and the heart was pushed to the right into the right pleural cavity to expose the common pulmonary venous chamber and posterior wall of the left atrium. A wide anastomosis was made between the common pulmonary venous chamber and the posterior wall of the left atrium. Both the cavae were snugged and the right atrium was opened. The ASD was closed using a Dacron patch in all patients. However, a fenestration was made in the Dacron patch in three patients who had raised PVR preoperatively. The right atrium was closed. The patient was rewarmed, the aortic cross-clamp was removed after de-airing the heart, and the aortic root was vented. Twelve patients were weaned off CPB with minimal inotropic support. The vertical vein was ligated in 14 patients, keeping a close watch on the hemodynamics. Three patients who had high preoperative PVR had difficulty in weaning from CPB. Of these three patients, two were weaned off from CPB with high inotropic support (dopamine 10 μg/kg/minute, dobutamine 1,012 g/kg/minute, and milrinone 0.5 μg/kg/minute). In the third patient, the aorta was again cross-clamped for removing the fenestrated ASD patch, following which we were able to wean off the patient from CPB on high inotropic support (dopamine 10 μg/kg/minute, dobutamine 10 μg/kg/minute, and milrinone 0.5 μg/kg/minute). There were no operative mortality and re-exploration. The mean CPB and cross-clamp time was 75 ± 12 minutes and 58 ± 9 minutes, respectively. The mean ventilatory support was 32 ± 5.3 hours. The mean inotropic support time was 2.1 ± 1.2 days. The mean intensive care unit (ICU) stay was 2.5 ± 0.8 days. Atrial fibrillation wasp was present in five (33.3%) patients in the early postoperative period which was managed with atrial overdrive pacing (Table 2). Three (20%) patients had pulmonary artery hypertensive crises postoperatively. Two patients were managed using prolonged ventilatory support and high inotropic support (dobutamine 10 μg/kg/minute, dopamine 10 µg/kg/minute, and milrinone 0.5 μg/kg/minute) in the postoperative period. The third patient went into low cardiac output syndrome despite high inotropic support. The patient developed severe metabolic acidosis and low urine output. Later, the patient had an acute renal shutdown for which peritoneal dialysis was started. The patient expired on the second postoperative day due to multiple organ dysfunction syndrome. There was no superficial or deep sternal wound infection in the postoperative period. The mean hospital stay was 8 ± 1.2 days. Echocardiography at the time of discharge showed no gradient across the anastomosis. Mild and moderate right ventricular dysfunction was present in four (26.67%) and two (13.3%) patients, respectively, for which oral sildenafil (0.3-2 kg) was administered postoperatively. On follow-up at one year, 12 patients were in functional class NYHA class I and II. On clinical examination, 12 (80%) cases had a systemic saturation of more than 96%. Two (13.3%) patients had a systemic oxygen saturation of 90 and 92%. On follow-up two-dimensional (echocardiography at the end of one year, there was no gradient across the anastomosis, and pulmonary artery pressure was normal in all patients.

## Discussion

The survival of patients with untreated supracardiac TAPVC is low. Almost 50% of these infants die within the first three months of life, and the mortality approaches 80% by the end of the first year [3]. In the remaining 20% of patients with supracardiac TAPVC, survival beyond infancy depends on the absence of pulmonary venous obstruction, dilated vertical vein, large and unobstructed ASD, and near-normal PVR [6]. On an extensive search of the literature, there is limited data available regarding these patients who survive beyond infancy. The presence of large and non-restrictive ASD helps in the prevention of the development of pulmonary arterial hypertension which facilitates the survival of these patients beyond infancy [7-10]. The postoperative outcomes of these patients depend on the absence of post-repair pulmonary venous obstruction [11]. In this study, there was no pulmonary venous flow obstruction preoperatively and a wide anastomosis was made between the common pulmonary venous chamber and the left atrium. The guide for the size of the anastomosis was the value of the mitral valve annulus indexed to the body surface area according to the nomogram chart. This facilitates unobstructed pulmonary venous flow, therefore, improving functional recovery and survival. Kirshbom et al. showed excellent long-term outcomes regarding survival, freedom from reintervention, and good overall functional ability in 100 infants who underwent repair of TAPVC [12]. Korbmacher et al. conducted a retrospective study in which 52 patients (of whom 21 were adolescent patients) who underwent TAPVC repair were analyzed. The mean follow-up was 10.7 years. The study concluded that postoperative rhythm disturbance is a predictor of poor surgical outcomes [13]. In this study, postoperative atrial fibrillation occurred in five (33%) patients and was managed medically. Reddy et al. conducted a prospective study among 26 patients who underwent TAPVC repair (mean age was 5.01 years). The mean ICU stay was 2.3 ± 0.87 days, and the mean hospital stay was 9.23 ± 2.34 days without major postoperative complications and early deaths. They concluded that in the absence of significant pulmonary vascular disease, surgical correction is indicated which results in regression of symptoms and pulmonary artery hypertension [14]. The present study also concludes that the absence of pulmonary venous obstruction and the presence of a large vertical vein and a large ASD have a favorable impact after surgical intervention with less inotropic and ventilator support and lower ICU and hospital stay. Therefore, surgical correction is advisable in such patients [15].

Limitations

The small study sample and retrospective design are the two major limitations of this study. As supracardiac TAPVC is an extremely rare anomaly and its presentation in the adolescent age group is rare, we came across only 15 patients who have undergone surgical repair within the duration of three years.

## Conclusions

Surgical repair of supracardiac TAPVC in adolescence has an excellent outcome. Survival of patients with supracardiac TAPVC until the age of adolescence depends on the presence of a dilated vertical vein and a large ASD facilitating unobstructed pulmonary venous flow. The aim of the surgical repair should be to create a wide anastomosis between the left atrium and the pulmonary venous chamber which should be bigger than the size of the mitral valve orifice indexed to the body surface area as it would amount to no or negligible anastomotic gradient postoperatively.
